# Supplementary material for: Phenotypic resistant single-cell characteristics under recurring ampicillin antibiotic exposure in Escherichia coli
Source: mSystems. 2024 Jun 26;9(7):e00256-24. doi: 10.1128/msystems.00256-24 (PMC11264686; doi:10.1128/msystems.00256-24)
Supplement: Supplemental Material — Additional details on experiments, conditions, and statistical analyses, as well as supplemental tables and figures. [file msystems.00256-24-s0001.pdf]

SI Phenotypic resistant single-cell characteristics under recurring ampicillin antibiotic exposure in *Escherichia coli*.

Silvia Kollerová[1], Lionel Jouvet[1], Julia Smelková [1], Sara Zunk-Parras[2], Alexandro Rodríguez-Rojas[2], Ulrich K. Steiner[1,2*]

MATERIALS AND METHODS

Bacteria cell culturing

All experiments and bacteria preparation were done in filtered minimum growth media supplemented with 0.4% Glucose and 0.2% Casamino Acids (throughout called M9). One liter of M9-media consisted of: 11.28 g M9 Salts 5x, 2 g Casein hydrolysate, 980 ml destilled water, 100 µl 0.1 M CaCl 2, 200 µl 1 M MgSO4, 20 mg 20% glucose. To prepare the media that was used for the constant nutrition flow during the microfluidic experiments, we added propidium iodide (PI) at 1 µg/ml final concentration.

To derive the initial stock suspension for the microfluidic (single-cell) experiments, we incubated a small aliquot of a frozen bacterial stock of MG1655 overnight in 25 ml M9 in an incubator shaker (C24, New Brunswick Scientific) at 37°C and 200 rpm. The next day we inoculated 50 µl of the overnight bacteria batch culture in 2x35 ml fresh M9 media and incubated at 37°C for ~4 hours until the bacterial suspension reached an optical density (OD600) of 0.4-0.6 (corresponding to the most reproductive exponential growth phase). After these 4 hours we washed the bacteria by initially centrifuging at 4000 rpm for 10 minutes at 37°C, discarding the supernatant, resuspending in 500 µl of fresh media, transferring the resuspension to an Eppendorf tube, centrifuged that for 1 minute at 10000 rpm, discarded the supernatant, and resuspended the bacterial pellet in 200 µl.

Microfluidic chip loading

We then transferred the 200 µl of concentrated bacterial suspension to a 1ml syringe and injected it into the main (feeding) channel of a microfluidic chip (1). For the cells to enter the side (growth) channels of the microfluidic chip, we centrifuged the injected microchip at 400 rpm (~168g) for 10 minutes at 37°C. Subsequently, we checked the loading under the microscope and reinjected bacteria for a second time, this time without centrifuging, to ensure adequate loading (loading efficiency >85%). Next, we placed the microchip under an inverted microscope (Nikon Ti) with a temperature-controlled incubator (Oeko-Labs), connected the in- and outflow tubes to the chip, and media was flown at a constant rate of 300 µl per hour using a peristaltic pump throughout the experiment. Prior to connecting the tubes, we sterilized the pump tubing together with autoclaved rust-free connectors in a plastic zip bag by 15-minute exposure in a UV sterilizer. All experiments were conducted under these conditions at a stable temperature of 37°C and the single-cell experiments were conducted under constant flow of media at a rate of 300 µl per hour.

The microfluidic chip the cells grew in contained four main laminar flow channels (feeding channels) and hundreds of dead-end side channels, in the latter the rod-shaped bacteria-cells grew. Only one focal cell, the one closest to the dead-end of the side channel, was tracked for its growth (cell elongation), size, time of each division, and death. Cell death was determined by propidium iodide (PI) that emits a strong fluorescent signal when the cell wall lysis—the moment the PI can enter the cell—and bonds to the cells' DNA. The cells loaded into the microfluidic chip came from an exponentially growing culture, and therefore the vast majority of cells loaded were in an exponential growth phase (2). Time between first centrifugation and attaching the loaded chip to the laminar flow was <40 min. During this time cells experienced high densities which might have impacted their growth.

Casting and mounting of the chip

We used polydimethylsiloxane (PDMS) (Sylgard Silicone Elastomer Base and Curing Agent mixed in 10:1 ratio) to caste the microfluidic chips. Specially prepared molds produced with Sigatec SA Master

(Smooth-Cast) were filled with PDMS and placed in an oven overnight (>12 hours) at 85°C. The next day we removed the casts from the molds under a clean air flow cabinet. The chip is designed with four main (feeding) channels, each holding 1200 side channels. Microfluidic inlets and outlets were punched by a sharpened gauge 22 needle at the start and end of each of the main (feeding) channels to later allow the flow of medium through the chip. Finally, we bonded the PDMS chip to a glass cover slide (24x60 mm) by treating the chip and slide for 30 seconds in an air plasma (plasma cleaner, PDC-002, Harrick Plasma), and immediately assembled the final microfluidic chip.

Microchip setup and phenotypic resistance validation protocol

To load cells into the chip we activated the chip surface by exposing it for 18 seconds to an air plasma, which makes the PDMS hydrophilic and allows injecting the media/20% PEG solution (400 µl M9- media + 100 µl PEG) inside the microchannels. We left the activated chip to incubate for at least 1 h before we loaded bacteria into the chip. PEG was used to prevent cell attachment to the chip and buildup of biofilms.

We flushed the in- and outflow connector tubes with media before connecting to the loaded chip, that was then mounted in a custom-designed microchip holder. The pump speed was set up to 300 µl per hour.

Time-lapse Imaging

Time-lapse images were taken at 4-minute intervals (15 frames/hour) and recorded by NIS Element AR software for data acquisition and microscope control. The fields of view were set up on each of the four main (feeding) channels of the chip, 15 fields of view per channel, together 60 for a whole microchip. Each field of view can record 23 dead-end-side (growth) channels with bacteria in them, that is, 1380 cells per chip could, theoretically, be tracked. However, the loading efficiency and the speed of the microscope table to change among field of views and autofocus does reduce this number. After defining the fields of view, two optical definitions (phase contrast and fluorescence)

were set up. Fluorescence was used to detect cell death by PI emitting a red signal at cell lysis (PI binding to the DNA strand).

Experimental treatment

We chose antibiotic concentrations similar to previously explored ranges, that spanned from sub-MIC to >30 times MIC, and exposed cells to antibiotics for periods that would cover ~three divisions under non-antibiotic conditions. Post-exposure recovery times were also chosen on previous results reporting on regained growth of phenotypically resistant cells within 3-4 hours post-treatment (3–5). We choose ampicillin as antibiotic, a β-lactamase that inhibits bacterial cell wall synthesis, because this antibiotic has been extensively used in previous persister studies (3).

For the experiments with recurrent exposure (both single-cell and cell culture experiments), each experiment ran for 18 hours. The first exposure period was 60-150 minutes past onset of the experiments, the second exposure period was 330-420 minutes past onset of the experiments, i.e. 180 minutes past the first exposure period. The last exposure period was 1080-1170 minutes past onset of the experiments, i.e. 660 minutes past the second exposure period. When switching between treatment, that is between media (with or without antibiotics), we added a tiny air bubble to the tube, that allowed us to accurately mark the time of change of the media in the chip and prevented potential diffusion of the two media in the tubing. For the single-cell experiments, concentrations of ampicillin during the exposure periods were set to 2, 4, 16, 24, 32, 64 or 128µg/ml. As comparison and reference, data from another experiment was added as a control group, where cells were never exposed to antibiotics (1). Individual cell numbers tracked were 0 µg/ml n~898 (data added from other experiment (1)), 2 µg/ml n~334, 4 µg/ml n~337, 16 µg/ml n~669, 24 µg/ml n~330, 32µg/ml n~347, 64 µg/ml n~230, 128µg/ml n~351. Note, not for all measures was data on all

cells available. For the experiments at the cell culture level, the concentrations of 0, 2, 4, 8, 16, 32, 64 or 128μg/ml were used, and in addition a negative control testing for potential contamination was done.

In addition to the single-cell experiments with 90-minute recurrent exposure periods we report on, we also ran two chips that had 30 minutes exposure periods. We do not report on these latter experiments as this short period did not lead to any antibiotic-exposure-triggered phenotypic effects and therefore are not presented.

Image analysis

After we collected the time-lapse images in ND format were exported to TIFF format and organized with customed programme TriFichier. The TIFF images were then analyzed with a custom written Visiopales programme (Opales Sarl, France). Afterwards, lab-written R scripts (6) checked for quality and errors were automatically corrected, see also (1). The final data matrix per experiment contained length of each cell at each 4-minute time point, fluorescence signal emitted from the PI to determine cell death (lysis) at each 4-minute time point, and time of division events of each cell throughout the experiment. From these matrixes we estimated in addition to size and division events, also time to death and growth rates.

Statistical analyses

Survival analysis

Kaplan-Meier survivorship curves (Fig. 2a) were computed with the survival package (survfit function; package [survival]) and compared among them with a Cox proportional hazard model in the R package [survival]. Cells that were still alive at the end of the experiment were noted to be right censored. Survival of 3496 cells were included in the analysis. A post-hoc test was then done to differentiate survival among the different concentrations of antibiotics by the pairwise_survdiff function of the survminer R package, applying a Holm p-value correction to account for multiple

testing (for test see Supplementary file 1A). The probability of death curves (Fig. 2b) were plotted with ggplot2 package and a loess smoothing (geometric_smooth function; package ggplot2). Competing GAM models (Table 1) are fitted with the restricted maximum likelihood method (REML), for those models a smoothing parameter for age (time since the start of the experiment) with a shrinkage version of a cubic regression spline as smoothing parameter (bs=cs) and the exposure concentration was explored. A null model (intercept only model), a model with only one factor either age or concentration, and a model with an additive or an interactive effect among age and concentration was compared. A Gaussian error structure and an identity link function was used for these GAM models. Probability of death curves (Fig. 5b) are plotted with a loess smoothing function with 95% CI (geometric_smooth; package ggplot2).

Growth rate analysis

We excluded a few events where growth rates were very small (<0.8) or very large (>1.4) as such rates are biologically not feasible. Growth rate curves (Fig. 4c) are fitted with a GAM (with a loess geometric_smooth function). Model comparison was done by comparing AIC among competing GAM models estimated with restricted maximum likelihood method (REML) and a Gamma error structure with an inverse link function. For those models, a smoothing parameter for age (time since the start of the experiment) with a shrinkage version of a cubic regression spline as smoothing parameter (bs=cs) and the exposure concentration was explored. A null model (intercept only model), a model with only one factor either age or concentration, and a model with an additive or an interactive effect among age and concentration was compared. Note that the smoothing does weaken the actual more pronounced drop in growth rates post-antibiotic exposure, we still present the smoothed curves to be more consistent with the other analyses presented.

Division rates

Division rates (Fig.4a) are fitted with a loess smoothing function (geometric_smooth; package ggplot2) using a binomial error structure with a logit link function. In very rare cases of more than

one division per 4-minute time step, that mainly happened when a filamentous cell divided into more than one cell at a time, we only considered this as a single division event to be able to use a binomial response variable (division yes/no) and an according error structure (binomial error structure and a logit link). Model comparison was done by comparing AIC among competing GAM models estimated with restricted maximum likelihood method (REML). For those models a smoothing parameter for age (time since the start of the experiment) with a shrinkage version of a cubic regression spline as smoothing parameter (bs=cs) and the exposure concentration was explored. A null model (intercept only model), a model with only one factor either age or concentration, and a model with an additive or an interactive effect among age and concentration was compared.

Size

Size curves (Fig. 4b) were fitted using a loess smoothing (geometric_smooth; package ggplot2) function. Model comparison was done by comparing AIC among competing GAM models estimated with restricted maximum likelihood method (REML). For those models a smoothing parameter for age (time since the start of the experiment) with a shrinkage version of a cubic regression spline as smoothing parameter (bs=cs) and the exposure concentration was explored. A null model (intercept only model), a model with only one factor either age or concentration, and a model with an additive or an interactive effect among age and concentration was compared. A Gaussian error structure and an identity link function was used for these GAM models. Note, smoothing functions (GAM) are highly sensitive at the extremes as partly illustrated by the diverse initial average cell sizes (Fig. 4b) of cells that came from the same parental population and that had not yet been exposed to antibiotics during the 60-minute lasting acclimation phase.

Comparing mortality among different growth categories

For cells exposed to concentrations at or above MIC we did a set of additional analyses. For these analyses we pooled cells across these concentration treatments.

Growth before death

We first tested whether cells that grew just before dying at high rates (>1.1), low rates (1.1-1.0), or were growth arrested (including shrinkage) (<1) differed in their age at death. For these tests we used GLM's with a gamma error structure and an inverse link function, age at death was the response variable and an intercept only model or a model that categorized cells into the three growth categories before death were compared using their AICs (Supplementary file 1B).

We then did a post-hoc test on the best model supported (GLM with Gamma error structure) to differentiate between pairs of growth categories in their age at death (Supplementary file 1C). For this, we used a Tukey test with the glht function in the mutlcomp R package.

Robustness of growth rates

To test for the robustness of growth rates we correlated growth rates at the current time t to the growth rate at time t+1, or t+3, or t+5. Curves (Fig. 6) were fitted using GAM models with the restricted maximum likelihood method, a smoothing parameter for Growth rate at time t with a shrinkage version of a cubic regression spline as smoothing parameter (bs=cs) and/or the concentration, including a model that accounts for the interaction of the two explanatory variables (growth at time t and time). Model comparison used the same GAM models with a Gaussian error structure and an identity link function.

Movie of mother machine during experiment

The time-lapse movie (Movie SI) shows one frame of view with 23 dead-end side channels. The focal cell is the cell most closely to the dead end (top of the channel). Below the side channels the main feeding channel with the laminar flow of media can be seen. Offspring cells are finally pushed out into this main feeding channel. The media also includes propidium iodide (PI), which emits at cell wall lysis (cell death) a red fluorescent signal. There recurring exposure of antibiotics (here 64µ/ml) is provided by the label.

Cell culture experiments

We inoculated 10 mL (1:100) from an overnight culture of *E. coli* MG1655 in the morning in fresh M9 medium and left cells to grow until the optical density of the culture at 600 nm was close to 0.2. At this point, we again inoculated the culture as before and added 200 µL to columns 1 to 8 of a 96-well plate. In the 9th column, we added 200 µL of M9 medium as a negative control. We then incubated this initial plate for 1 h at 37°C and vigorous shaking (Supplemental Figure S5).

After this incubation time, we took a sample. For this, we transferred 50 µL of each column individually to a new multi-well plate, where the first column was completely empty and the rest of them until the column 6 contained 180 µL of NaCl for serial dilutions before we plated the samples on LB-agar plates. We used a total of eight multi-well plates for these serial dilutions, each plate for one column of the initial plate, and therefore eight LB-agar plates resulted from each sampling with 8 replicates (rows of plates).

Next, we exposed bacteria to ampicillin as for the single-cell experiments. From the initial plate, we transferred 100 µL of each well to a V-bottom-96-well plate containing 100 µL of serial dilutions of the antibiotic. The starting concentration at which bacteria were exposed was 128 µg/mL and the lowest, 2, passing through 64, 32, 16, 8 and 4 (note the single-cell experiments did not have 8 µg/mL exposure but 24 µg/mL). Each concentration was represented by a column in the multi-well plate, and every row constituted a replicate, obtaining a total of 8 replicates per concentration. The two last columns were always left as positive and negative controls, respectively.

Once we had prepared the plate, we incubated it for 90 min in the same conditions as above. When this exposure time passed, we took another sample as described before and plated a serial dilution on agar plates. We removed the antibiotic in the V-bottom-96-well plate by centrifuging 10 min at 4000 g and aspired the supernatant. We then resuspended cells in 200 µL of fresh M9 and incubated them for 3 h. After the recuperation time, we took another sample (serial dilution and plating on agar plates), and the bacteria were exposed again to decreasing concentrations of ampicillin as

described before. The exposure time was again 90 min, after which we took the third sample, the antibiotic was again removed and a recovery period of 11 h in fresh medium followed.

Following these 11 h, we took a new sample, and transferred the bacteria to a new set of plates with decreasing antibiotic concentrations as we had previously done. We incubated one last time the cell cultures for 90 min. We then extracted the last sample after this final exposure time.

We incubated all the LB-agar plates at 30°C and counted the colonies (Colony Forming Units) after 16 h of incubation.

Cell culture experiments constant exposure to antibiotics

To track cell culture density at constant antibiotic exposure, the initial 8 replicates for each concentration of the antibiotic exposure were incubated at 37°C and shaking in a 96-well-plate and the optical density (OD600) was measured every 20 minutes for 24 h in a plate reader Synergy H1 (Biotek, Germany). Fig. 7b the OD curves are fitted with a loess smoothing function with 95% CI (geometric_smooth with a span of 0.1; package ggplot2).

STATISTICS TABLES & FIGURES

1A)

Table A: Comparison of survival curves among groups of cells exposed to the 8 different levels of antibiotic concentrations (Fig. 2a). Testing is based on a Likelihood ratio test for the overall model and on Cox proportional hazard rates for among survival curves with 0 µg/ml as reference curve. Cells that were alive at the end of the experiments are noted to be right censored (n= 276). Likelihood ratio test=660.8 on 7 df, p< 0.0001, n= 3496, number of events= 3220.

**ANTIBIOTICS CONCENTRATION**	**COEFFICIENT (±SE)**	**Z**	**P**
**2**	-0.023(±0.071)	-0.32	0.75
**4**	0.917(±0.067)	13.65	<0.0001
**16**	0.916(±0.055)	16.68	<0.0001
**24**	1.047(±0.068)	15.39	<0.0001
**32**	0.947(±0.066)	14.13	<0.0001
**64**	1.12(±0.077)	14.59	<0.0001
**128**	0.988(±0.066)	14.89	<0.0001

Post-hoc pairwise comparison testing survival curve differences among groups of cells exposed to the 8 different levels of antibiotic concentrations (Fig. 2a). Pairwise testing is based on a log rank test with p-value adjustment based on Holm. Cells that were alive at the end of the experiments are noted to be right censored. Values shown are pairwise p-values.

**ANTIBIOTICS CONCENTRATION**	**0**	**2**	**4**	**16**	**24**	**32**	**64**
**2**	1.000	-	-	-	-	-	-
**4**	<2e-16	<2e-16	-	-	-	-	-
**16**	<2e-16	<2e-16	1.000	-	-	-	-
**24**	<2e-16	<2e-16	0.866	0.390	-	-	-
**32**	<2e-16	<2e-16	1.000	1.000	1.000	-	-
**64**	<2e-16	<2e-16	0.173	0.047	1.000	0.456	-
**128**	<2e-16	<2e-16	1.000	1.000	1.000	1.000	1.000

1B)

Table B: Growth dependent mortality comparison

	**DF**	**AIC**
**Age at death ~ Growth Category**	**4**	**22645.3**
**Age at death ~ 1**	2	22671.1
Best supported model highlighted in bold. Age at death assigned to last growth rate. Only cells of groups exposed to >= 4 µg/ml included in analysis. Model comparison for GLM with Age at death as response variable, the growth categories (High, Low, Shrinkage) as explanatory variable, or a null model (intercept only model), with a gamma error structure.		

1C)

Table C: Pairwise comparison among different growth categories for age at death

Pairwise comparison	Estimate	Std. Error	z value	Pr(>|z|)
Slow - Fast	-0.0022818	0.0004944	-4.615	<0.001
Shrink - Fast	-0.0007707	0.0006717	-1.147	0.4801
Shrink - Slow	0.0015111	0.0005921	2.552	0.0281
Adjusted p-values reported — single-step method				

Fig. S1:

QQ-plots to evaluate normally distributed residuals using the R package DHARMa for (a) Division rates (GAM binomial)(Fig. 4a), (b) Size (Gam Gamma (identity link))(Fig. 4b), (c) Size (Gam Gamma (identity link))(Fig. 4c). Size and growth data (b & c) show some overdispersion, which likely arises from filamentation of some cells (continued growth without division).

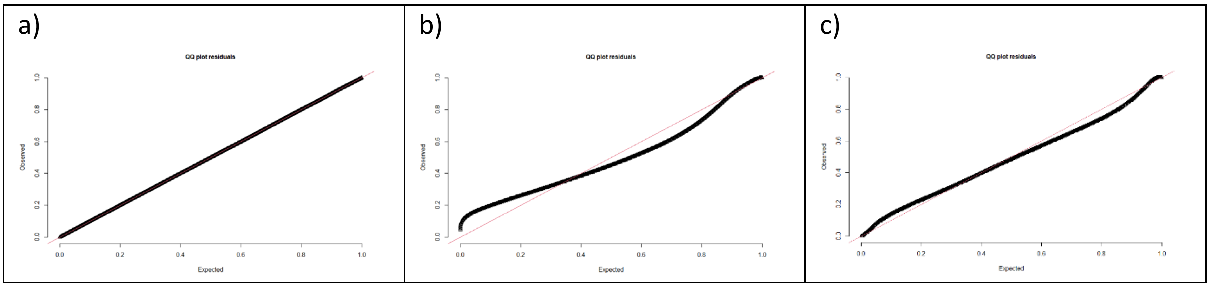

Table D:

In order to evaluate the evolutionary potential for fixed antibiotic resistance, we compare division numbers among susceptible (N= 1521 cells dying before 330 min, before onset of the second exposure period) and phenotypic resistant cells (N=743 cells dying after 330 min, after onset of the second exposure period) exposed to MIC or higher, and relate these to their number of division over the course of the experiment. Note, this estimation is conservative as we are not considering right censoring and some phenotypic resistant cells still being alive at the end of the experiment and would continue to divide. The findings show that phenotypic resistant cells make only 1/3 of the cells are responsible for ~2/3 of all divisions. Therefore, each phenotypic cell has a four times higher chance of a genetic fixed mutation to occur.

Concentration	Number Susceptible	Number Phenot. Resistant	%Phenot. Resistant	Lifetime reproductive output (Number Divisions Susceptible)	Lifetime reproductive output (Number Division Phenot. Resistant)	%Phenot. Resistant contribution to LRO
4	209	128	38	840	1956	70
16	436	233	35	2453	3806	61
24	237	93	28	1106	1437	56
32	225	122	35	1098	2102	65
64	167	63	27	541	884	62
128	247	104	30	1117	1730	61
Total	1521	743	33	7155	11915	62

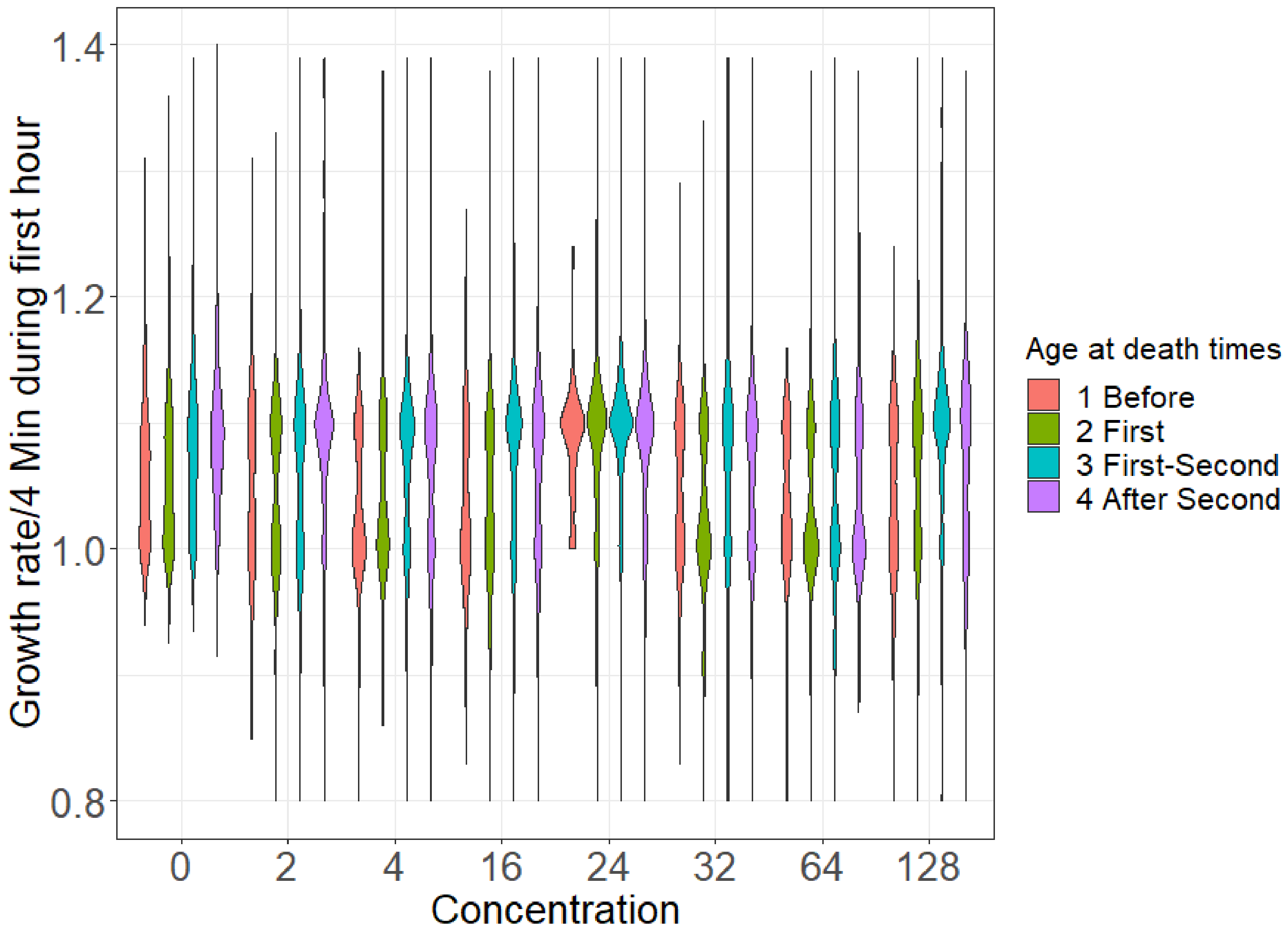

Fig. S2: Comparison of growth rates prior to the first exposure period. The figure shows growth rates during the first hour of the experiment for cells that were later exposed to different concentrations of antibiotics and the different categories (colours) refer to groups of cells that died either before the first exposure to antibiotics (red), that died during the first exposure period (green), that died between the first and second antibiotic exposure period (blue) or that died after the second antibiotic exposure period (violet). Cells that were later phenotypic resistant did not show distinct growth rates prior to exposure.

1E)

Table E: Model comparison among growth rates prior to first exposure of cells later exposed to different antibiotic concentrations and groups of cells dying during or surviving exposure periods.

	DF	AIC
**Growth rate prior ~ Concentration*Age at death category**	**33**	**-108752**
**Growth rate prior ~ Concentration+Age at death category**	12	-108505
**Growth rate prior ~ Concentration**	9	-107890
**Growth rate prior ~ Age at death category**	5	-107724
**Growth rate prior ~ 1**	2	-107009

Best supported model highlighted in bold. Model comparison for linear model with Growth rate prior to first exposure as response variable, the Age at death categories (Before first exposure, during first exposure, between first and second exposure, and after second exposure) as well as the later exposure concentrations as explanatory variable. Compared were all single, additive, interactive effects and a null model (intercept only model).

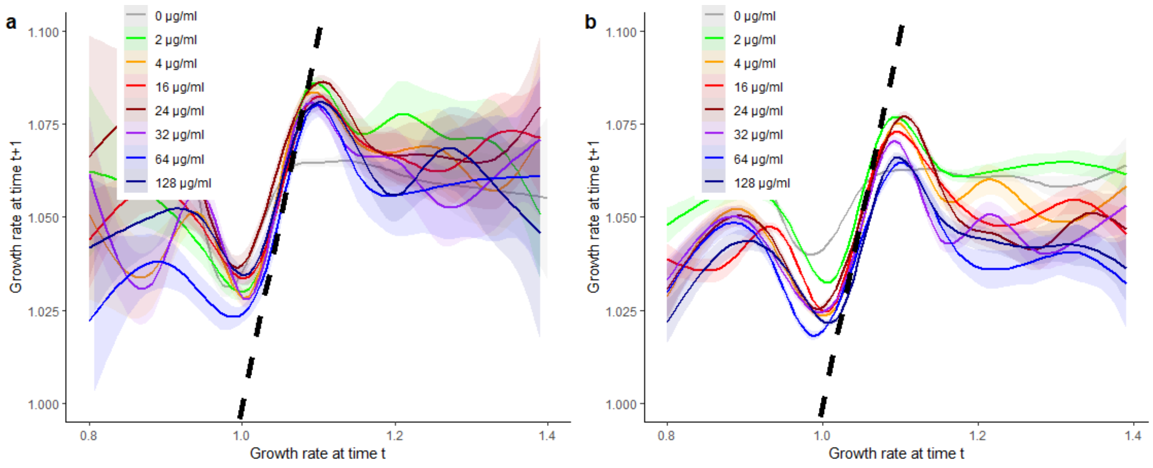

Fig. S3: Correlation in cell growth rates at a given time t, compared to growth rates at time t+1 (4 minutes later) for growth periods before the onset of the first antibiotic exposure (a), and after the onset of antibiotic exposure(b), separated out into populations recurrently exposed to different levels of antibiotics (as described in the main experiment). All growth rate correlations are fitted with a GAM with 95% CI. The dashed black line illustrates the direct correlation among time points. The combined data for both periods is shown in Fig. 6a of the main text. Model comparisons among patterns in panel (a) and (b) are given in Table D.

Table F: Model comparison among competing GAM models, for correlation of current growth rates to growth rates at time t+1 for two periods, i) before first exposure and ii) after first exposure (see Fig. S3).

MODEL	DF	AIC
Growth_t+1~Concentr. + S(Growth by Concentr)	80.6	-1005662
**Concentr. * period + S(Growth by interaction(Concentr*period)**	**155.0**	**-1006618**

Best supported model is highlighted in bold. GAM models are fitted with the restricted maximum likelihood method, a smoothing parameter for Growth rate at time t. Note as a shrinkage version of a cubic regression spline as smoothing parameter (bs=cs) is not supported for interacting models, such smoothing parameter was not specified. The concentration is included in all models. Additive or interactive effects of period (Growth correlation before or after onset of antibiotic exposure) is included in the model comparison. Growth rates are fitted with a Gaussian error structure and an inverse link function.

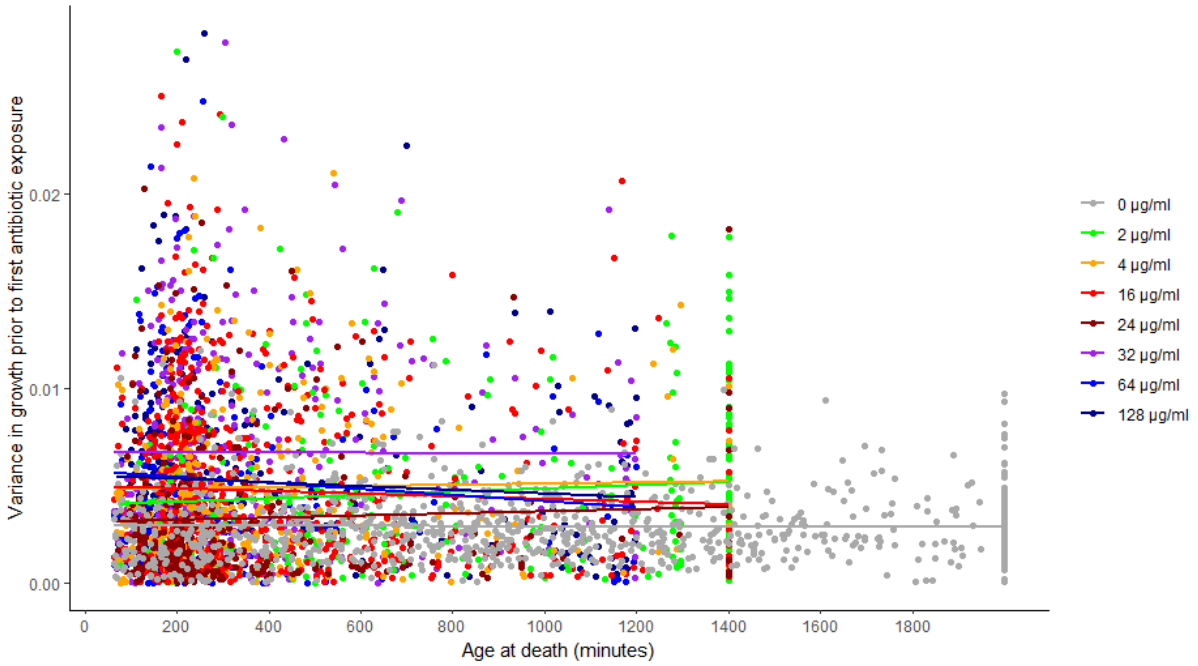

Fig. S4: Variance in individual cell growth prior to the first exposure to antibiotics (variance in growth in the first 60 min of experiment) plotted against age at death. Each data point resembles one cell. Linear fits for each concentration of antibiotic are shown by the colored lines. Overall, there was no clear pattern of decreased variance prior to antibiotic exposure (more robust growth early in life) resulting in increased survival probabilities (negative slopes). This suggests that variance (growth robustness) early in life cannot be used to predict likelihood of cells switching to phenotypic resistance later in life. Statistical model comparisons suggest that a model that accounts for age at death and concentration (AIC -28518 df=17) is not better supported than one that does not consider age at death (AIC -28523, df=9).

Table G: Model comparison among competing linear models, for log(CFU counts) (response variable) and time (as a factor) and concentration as explanatory variables (see Fig. 7a main text).

MODEL	DF	AIC
**log(CFUcounts)~TimeFactor*Concentr**	**49**	**1199**
log(CFUcounts)~TimeFactor+Concentr	14	1908
log(CFUcounts)~Concentr	9	1967
log(CFUcounts)~TimeFactor	7	2313
log(CFUcounts)~1 (intercept only)	2	2327

Best supported model is highlighted in bold. The model is fitted with a Gaussian error structure. To reduce overdispersion and correct for heteroscedasticity, we transformed CFU counts to log(CFU counts) as in Fig. 7a main text. We also added 1 to all CFU counts as log(0) is not defined. We used Time as a factor and not a continuous variable to account for the different episodes of antibiotic exposure. The Summary statistics of the best supported model (Coefficients from R and associated post hoc testing) are shown in Table H.

Table H: Summary statistics of best supported model for CFU counts from Table G

term	estimate	std.error	statistic	p.value
(Intercept)	14.9406188	0.3835835	38.9501046	0.0000000
Conc2	-1.7856893	0.5424690	-3.2917811	0.0011013
Conc4	0.2338003	0.5424690	0.4309928	0.6667500
Conc8	0.5081288	0.5424690	0.9366961	0.3495877
Conc16	-0.1455940	0.5424690	-0.2683913	0.7885628
Conc32	-0.1829821	0.5424690	-0.3373134	0.7360913
Conc64	-0.3166284	0.5424690	-0.5836801	0.5598272
Conc128	0.7338544	0.5424690	1.3528042	0.1770278
TimeFact1	0.8531083	0.5424690	1.5726395	0.1167434
TimeFact2	2.8819627	0.5424690	5.3126768	0.0000002
TimeFact3	2.9140279	0.5424690	5.3717866	0.0000001
TimeFact4	5.4362103	0.5424690	10.0212360	0.0000000
TimeFact5	5.0133307	0.5424690	9.2416900	0.0000000
Conc2:TimeFact1	0.9955218	0.7671671	1.2976597	0.1952945
Conc4:TimeFact1	-0.8805422	0.7671671	-1.1477842	0.2518742
Conc8:TimeFact1	-2.3257883	0.7671671	-3.0316581	0.0026212
Conc16:TimeFact1	-2.2862483	0.7671671	-2.9801179	0.0030914
Conc32:TimeFact1	-2.9949314	0.7671671	-3.9038842	0.0001144
Conc64:TimeFact1	-4.5368340	0.7671671	-5.9137496	0.0000000
Conc128:TimeFact1	-8.3570485	0.7671671	-10.8933880	0.0000000
Conc2:TimeFact2	1.8409919	0.7671671	2.3997275	0.0169512
Conc4:TimeFact2	-0.6586534	0.7671671	-0.8585528	0.3911994
Conc8:TimeFact2	-1.4039348	0.7671671	-1.8300249	0.0681320
Conc16:TimeFact2	-1.4425635	0.7671671	-1.8803773	0.0609215
Conc32:TimeFact2	-2.4230713	0.7671671	-3.1584662	0.0017299
Conc64:TimeFact2	-5.8661281	0.7671671	-7.6464806	0.0000000
Conc128:TimeFact2	-9.6465024	0.7671671	-12.5741873	0.0000000
Conc2:TimeFact3	2.0045644	0.7671671	2.6129438	0.0093800
Conc4:TimeFact3	-0.3000553	0.7671671	-0.3911212	0.6959556
Conc8:TimeFact3	-1.6004552	0.7671671	-2.0861886	0.0377153
Conc16:TimeFact3	-1.6896189	0.7671671	-2.2024133	0.0283149
Conc32:TimeFact3	-3.5014889	0.7671671	-4.5641804	0.0000070
Conc64:TimeFact3	-8.3316768	0.7671671	-10.8603160	0.0000000
Conc128:TimeFact3	-17.9255880	0.7671671	-23.3659508	0.0000000
Conc2:TimeFact4	1.9253409	0.7671671	2.5096761	0.0125540
Conc4:TimeFact4	-0.2332233	0.7671671	-0.3040058	0.7613116
Conc8:TimeFact4	-0.3917906	0.7671671	-0.5106979	0.6098980
Conc16:TimeFact4	-0.0779462	0.7671671	-0.1016026	0.9191327
Conc32:TimeFact4	-0.3268025	0.7671671	-0.4259861	0.6703908
Conc64:TimeFact4	-6.9577636	0.7671671	-9.0694242	0.0000000
Conc128:TimeFact4	-20.0511110	0.7671671	-26.1365637	0.0000000
Conc2:TimeFact5	1.7360706	0.7671671	2.2629629	0.0242758
Conc4:TimeFact5	-0.2169994	0.7671671	-0.2828580	0.7774598
Conc8:TimeFact5	-0.3159753	0.7671671	-0.4118729	0.6806952
Conc16:TimeFact5	-0.8141018	0.7671671	-1.0611793	0.2893706
Conc32:TimeFact5	-6.3427546	0.7671671	-8.2677618	0.0000000
Conc64:TimeFact5	-7.8878250	0.7671671	-10.2817565	0.0000000
Conc128:TimeFact5	-20.6878038	0.7671671	-26.9664910	0.0000000

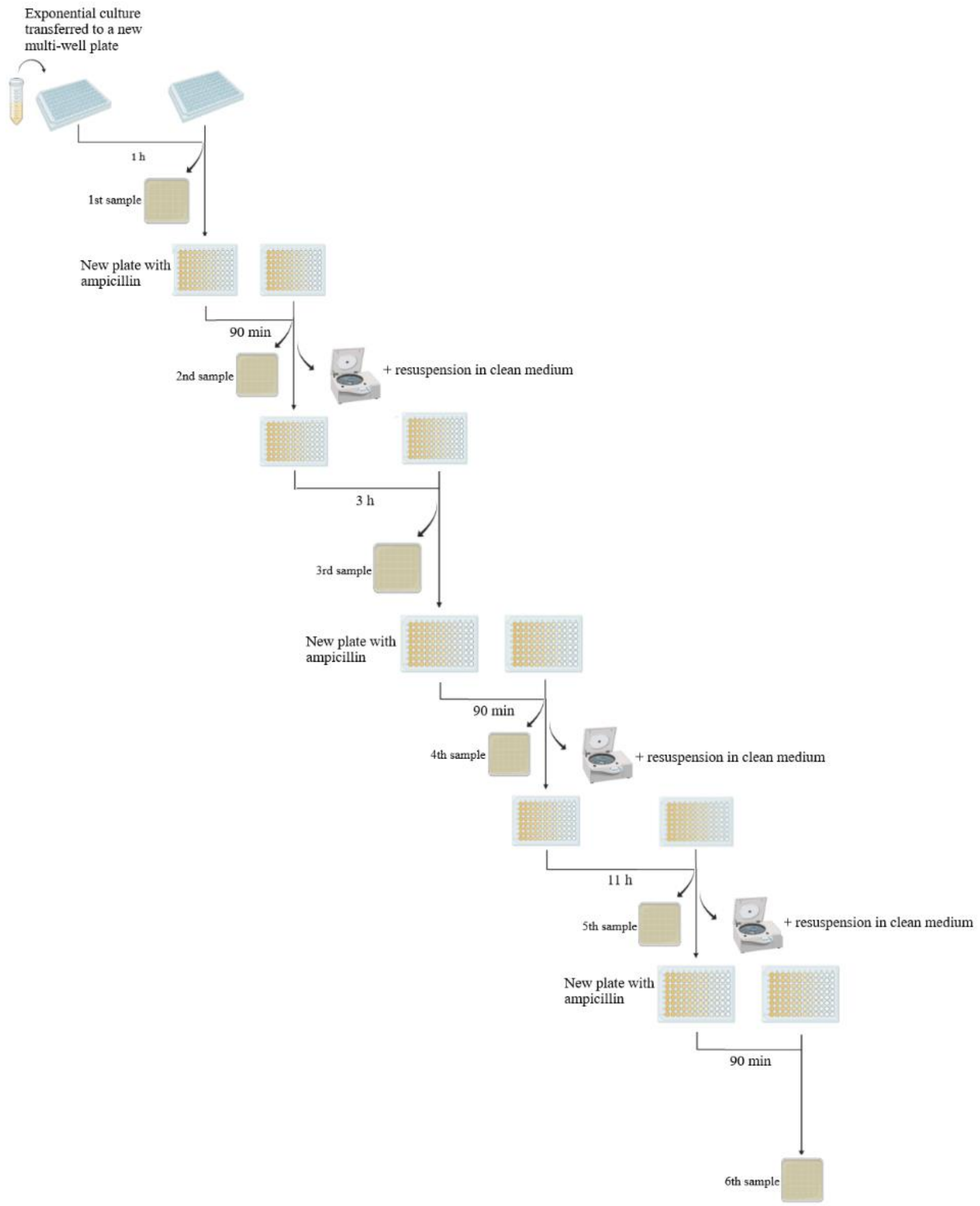

Fig. S5: Workflow for the cell culture experiments. The cell culture in exponential phase was added into a 96-multi-well plate and incubated for 1 h at 37°C and vigorous shaking. After this incubation time, a sample was taken from each well, serially diluted and plated in a LB-agar plate. Then, from the original multi-well plate, 100 µl were transferred to a new plate with the serial dilutions of the antibiotic, which was incubated again for 90 min. Each of the new plates with ampicillin that replaced the old one is highlighted in green. When the incubation time passed, another sample was taken, the plate was centrifuged to remove the antibiotic, cells were resuspended in new medium and a recovery period of 3 h followed. After this, a new sample was collected, the cells were again exposed to the antibiotic as described before and were left for incubation during 90 min. Once more, another sample was plated, and the antibiotic was removed to let the cells recover for 11 h. At the end, a new sample was collected, cells were exposed for the last time to the antibiotic for 90 min and the last sample was taken after this.
