# Supplementary material for: Phenotypic resistant single-cell characteristics under recurring ampicillin antibiotic exposure in Escherichia coli
Source: mSystems. 2024 Jun 26;9(7):e00256-24. doi: 10.1128/msystems.00256-24 (PMC11264686; doi:10.1128/msystems.00256-24)
Supplement: Legend — Movie S1 legend. [file msystems.00256-24-s0002.pdf]

SI Movie

The Movie (SI Movie) shows an exemplar view of the mother machine (one frame) with recurring exposure to antibiotics at 64µg/ml. Single cells are growing in the side channels, in each channel the cell closest to the dead end of the side channel is the one tracked (see also Fig. 1 main text). Red fluorescent cells are dying cells with compromised cell walls.
